# Hybrid Nanofluids Flows Determined by a Permeable Power-Law Stretching/Shrinking Sheet Modulated by Orthogonal Surface Shear

**DOI:** 10.3390/e23070813

**Published:** 2021-06-25

**Authors:** Natalia C. Roşca, Ioan Pop

**Affiliations:** Department of Mathematics, Faculty of Mathematics and Computer Science, Babeş-Bolyai University, 400084 Cluj-Napoca, Romania; popm.ioan@yahoo.co.uk

**Keywords:** hybrid nanofluid, power law, stretching/shrinking sheet, heat transfer, critical points, numerical methods

## Abstract

The present paper studies the flow and heat transfer of the hybrid nanofluids flows induced by a permeable power-law stretching/shrinking surface modulated orthogonal surface shear. The governing partial differential equations were converted into non-linear ordinary differential equations by using proper similarity transformations. These equations were then solved applying a numerical technique, namely bvp4c solver in MATLAB. Results of the flow field, temperature distribution, reduced skin friction coefficient and reduced Nusselt number were deduced. It was found that increasing mass flux parameter slows down the velocity and, hence, decreases the temperature. Furthermore, on enlarging the stretching parameter, the velocity and temperature increases and decreases, respectively. In addition, that the radiation parameter can effectively control the thermal boundary layer. Finally, the temperature decreases when the values of the temperature parameter increases. We apply similarity transformation in order to transform the governing model into a system of ODEs (ordinary differential equations). Numerical solutions for particular values of involved parameters are in very good agreement with previous calculations. The most important and interesting result of this paper is that for both the cases of shrinking and stretching sheet flows exhibit dual solutions in some intervals of the shrinking and stretching parameter. In spite of numerous published papers on the flow and heat transfer over a permeable stretching/shrinking surface in nanofluids and hybrid nanofluids, none of the researchers studied the present problem. Therefore, we believe that the results of the present paper are new, and have many industrial applications.

## 1. Introduction

During the last few years, hybrid nanofluids appeared as an extension of nanofluids and are believed to improve their thermophysical and rheological characteristics and also heat transfer attributes. Suresh et al. [1] supervised an analysis from an experimental point of view on the synthesis and characterization of ${\mathrm{Al}}_{2}O_{3}–\mathrm{Cu}/H_{2}O$ nanoparticles for various concentrations: 0.1%; 0.33%; 0.75%; 1%; and 2%. Huminic and Huminic [2] have affirmed a review paper that the results concerning the thermo-physical properties and the heat transfer and flow characteristics of hybrid nanofluids are used in various heat exchangers and energy applications. Hybrid nanofluid is invented by combining a base fluid with a mixture or composite form of suspended dissimilar nanoparticles. This kind of nanofluid was found to have great efficiency in terms of its thermophysical properties. The reviews on hybrid nanofluids were briefly discussed in the references [3,4]. Mabood et al. [5] highlighted the use of Fe_3_O_4_-graphene/water hybrid nanoliquid in three-dimensional unsteady boundary layer flow with magnetic field and non-linear radiation. Devi and Devi [6] predicted the thermophysical properties of hybrid nanofluid by using the correlations which have been validated with the existing experimental data. Moreover, the correlations of hybrid nanofluids in Takabi and Salehi [7] were also practical and considered by few researchers. Later, Khashi’ie et al. [8] used both types of thermophysical properties (Devi and Devi [6] and Takabi and Salehi [7]). Experimental and numerical results presented in many papers indicate that the hybrid nano-sized particle fluids will enhance the heat transfer in heat exchangers significantly, but research is still needed regarding the study of distinct combinations of nanoparticles, their mixing ratio, the stability of the hybrid nanofluids, and the understanding the mechanisms which contribution to the heat transfer enhancement. Devi and Devi [6] engaged a numerical study of the Cu-${\mathrm{Al}}_{2}O_{3}$/water hybrid nanofluid in two-dimensional flow over a sheet which is stretched. It was found that by choosing various and suitable quantities of nanoparticles the best choice heat transfer rate of hybrid nanofluid will be obtained. Since the paper by Devi and Devi [6] was published, many researchers have studied the hybrid nanofluids past surfaces that are stretched or shrunk, such as Rostami et al. [9], Waini et al. [10,11], Khashi’ie et al. [12], Zainal et al. [13], to mention just a few authors. We can also mention the following published papers suggested by reviewers: Rahman et al. [14], Shafiq [15,16,17], Sanni et al. [18], Khan et al. [19], Khan [20] and Makinde [21], Mabood et al. [5]. The heat transfer applications of hybrid nanofluids are: industrial cooling applications; smart fluids (due to the lack of abundant sources of clean energy and widespread dissemination of battery operated devices, such as cell phones and laptop it is essential to use nanofluids as a smart fluid; biomedical applications; cancer therapeutics (this initiative involves the use of iron-based nanoparticles as delivery vehicles for drugs or radiation in cancer patients); liquid cooling of computer processors due to their high thermal conductivity. emulsions, spray pyrolysis; and thermal spraying, etc.

The boundary layer flows driven by stretching/shrinking sheets have been investigated by a lot of people due to their many applications. The heat transfer and flow characteristics generated by stretching/shrinking surfaces appear widely in engineering application processes (see Fisher [22]). Therefore, in the present paper, we intend to analyze the hybrid nanofluid flows induced by a permeable power-law stretching/shrinking surface modulated by orthogonal surface shear. The influences of various parameters over the main physical quantities of interest are provided in figures and are discussed in detail.

## 2. Mathematical Model

**Figure 1:**
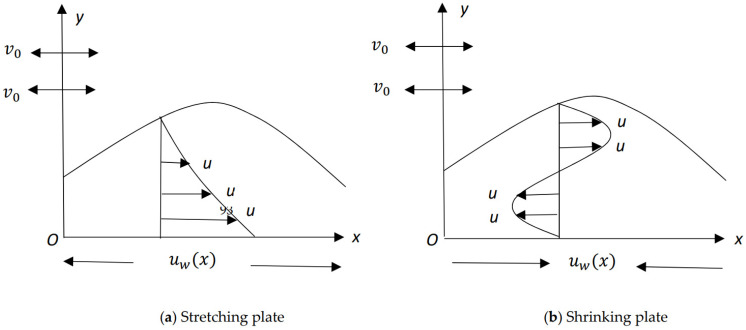
Physical scheme the proposed problem.

This paper extends the zero-pressure-gradient boundary-layer solutions for power-law stretching/shrinking sheet studied by Banks [23] to include an orthogonal power-law shearing motion with equal exponents to the important case of hybrid nanoparticle-sized fluids. Figure 1 presents the schematic of the problem, in which (x, y) axes are taken in the plane of the sheet and z−axis is normal directions to the surface of the sheet, the flow being in the region z≥0. We suppose that the velocity of the stretching/shrinking sheet is $u_{w}\left(x\right)$ and the mass flux velocity is $w_{w}\left(x,y,0\right)$, with $w_{w}\left(x,y,0\right)<0$ for suction case and $w_{w}\left(x,y,0\right)>0$ for injection case. We also assume that the constant surface temperature is $T_{w}$, and $T_{\infty }$ is the temperature of the ambient fluid.

Based on these hypotheses and using hybrid nanofluid model suggested by Takabi and Salehi [7], the governing conservation equations of continuity, streamwise momentum, the spanwise momentum and energy equations, are expressed in the Cartesian coordinates (x, y, z) as (see Weidman [24]; Devi and Devi [6]):

(1) $$\frac{\partial \;u\;}{\partial \;x}+\frac{\partial \;v\;}{\partial \;y}+\frac{\partial \;w\;}{\partial \;z}=0$$

(2) $$u\;\frac{\partial \;u}{\partial \;x}+v\;\frac{\partial \;u}{\partial \;y}+w\;\frac{\partial \;u}{\partial \;z}=\frac{\mu_{hnf}}{\rho_{hnf}}\;\frac{\partial^{2}\;u}{\partial \;z^{2}}$$

(3) $$u\;\frac{\partial \;v}{\partial \;x}+v\;\frac{\partial \;v}{\partial \;x}+w\;\frac{\partial \;v}{\partial \;z}=\frac{\mu_{hnf}}{\rho_{hnf}}\;\frac{\partial^{2}v}{\partial \;z^{2}}$$

(4) $$u\;\frac{\partial \;T}{\partial \;x}+v\;\frac{\partial \;T}{\partial \;y}+w\;\frac{\partial T}{\partial \;z}=\frac{k_{hnf}}{{\left(\rho \;C_{p}\right)}_{hnf}}\;\frac{\partial^{2}T}{\partial \;z^{2}}$$

subject to the boundary conditions:

(5) $$\begin{array}{r} \begin{array}{c} u\left(x,y,z\right)=u_{w}\left(x,y,z\right)\;\lambda =a\;x^{n}\lambda ,\;\;\;v\left(x,y,z\right)=v_{w}\left(x,y,z\right)=bx^{n}\\ w=w_{w}\left(x,y,z\right),\\ T=T_{w} \end{array})\;\mathrm{at}\;z=0\\ u\left(x,y,z\right)\to 0,\;v\left(x,y,z\right)\to 0,\;w\left(x,y,z\right)\to 0,\;T\to T_{\infty }\;\mathrm{as}\;z\to \infty \end{array}\}$$

Here u(x,y,z), v(x,y,z) and w(x,y,z) represent the components of the velocity along (x,y, z) axes, T is the temperature of the hybrid nanofluid, a is a positive constant having dimension $t^{-1},$ where a describes the stretching/shrinking strength of the sheet and λ is the stretching/shrinking parameter of the sheet which is constant, with λ>0 for the stretching case, λ<0 for the shrinking case and λ=0 for the fixed sheet, respectively, and n is the power-law stretching/shrinking parameter not being required to be an integer number.

Furthermore, $\mu_{hnf}$ represents the dynamic viscosity, $\rho_{nhf}$ represents the density, $k_{hnf}$ represents the thermal conductivity and ${\left(\rho \;C_{p}\right)}_{hnf}$ represents the heat capacity of the hybrid nanoparticles fluid, calculated accordingly to Takabi and Salehi [7].

(6) $$\begin{array}{c} \mu_{hnf}=\mu_{f}{\left(1-\phi_{Al_{2}O_{2}}-\phi_{Cu}\right)}^{-2.5}\\ \rho_{hnf}=\phi_{Al_{2}O_{3}}\rho_{Al_{2}O_{3}}+\phi_{Cu}\rho_{Cu}+\left(1-\phi_{hnf}\right)\rho_{f}\;\\ \frac{k_{hnf}}{k_{f}}=\left\{\frac{\phi_{Al_{2}O_{3}}k_{Al_{2}O_{3}}+\phi_{Cu}k_{Cu}}{\phi_{Al_{2}O_{3}}+\phi_{Cu}}+2k_{f}+2\left(\phi_{Al_{2}O_{3}}k_{Al_{2}O_{3}}+\phi_{Cu}k_{Cu}\right)-2\left(\phi_{Al_{2}O_{3}}+\phi_{Cu}\right)k_{f}\right\}\;\times \\ {\left\{\frac{\phi_{Al_{2}O_{3}}k_{Al_{2}O_{3}}+\phi_{Cu}k_{Cu}}{\phi_{Al_{2}O_{3}}+\phi_{Cu}}+2k_{f}-\left(\phi_{Al_{2}O_{3}}k_{Al_{2}O_{3}}+\phi_{Cu}k_{Cu}\right)+\left(\phi_{Al_{2}O_{3}}+\phi_{Cu}\right)k_{f}\right\}}^{-1}\\ {\left(\rho C_{p}\right)}_{hnf}=\phi_{Al_{2}O_{3}}{\left(\rho C_{p}\right)}_{Al_{2}O_{3}}+\phi_{Cu}{\left(\rho C_{p}\right)}_{Cu}+\left(1-\phi_{hnf}\right){\left(\rho C_{p}\right)}_{f} \end{array}\}$$

Here ϕ is the volume fraction of the nanoparticles (ϕ = 0 corresponding to a normal fluid), $\phi_{Al_{2}O_{3}}$ denotes Al_2_O_3_ particles and $\phi_{Cu}$ denotes Cu particles), $\rho_{f}$ and $\rho_{s}$ represents the densities of the base fluid and the hybrid nano-sized particles, respectively, *k_f_* and *k_s_* represents the thermal conductivities of the base fluid and the hybrid nano-sized particles, respectively, ${\left(\rho C_{p}\right)}_{f}$ and ${\left(\rho C_{p}\right)}_{s}$ represents the heat capacitance of the base fluid and the hybrid nano-sized particle, and $C_{p}$ is the heat capacity at constant pressure. The above relations are correct and realistic. They are determined starting from a physical hypothesis, and they agree with the mass and energy conservation. Table 1 presents in detail the physical properties of the base fluid (water) and nano-sized particles that are of great interest for us.

According to Weidman [24], the next variables which are dimensionless are introduced:

(7) $$\begin{array}{c} \;u\left(x,y,\eta \right)=a\;x^{n}f^{\prime }\left(\eta \right),\;\;\;v\left(x,y,\eta \right)=b\;x^{n}\;g^{\prime }\left(\eta \right),\\ w=\;-\frac{\sqrt{a\;\nu_{f}}}{\mu_{f}}\left\{n\;x^{n-1}\;f\left(\eta \right)+x^{n}\frac{\sigma_{x}}{\mu_{f}}\;\left[\eta \;f^{\prime }\left(\eta \right)-f\left(\eta \right)\right]\right\}\\ \theta \left(\eta \right)=\frac{T-\;T_{\infty }}{T_{w\;}-\;T_{\infty }},\;\;\;\;\;\eta =z\;\sqrt{a/\nu_{f}}\;\sigma \left(x\right) \end{array}\}$$

Thus,

(8) $$w_{w}\left(x,0\right)=\frac{\sqrt{a\;\nu_{f}}}{\mu_{f}}\;\left(n\;x^{n-1}-x^{n}\frac{\sigma_{x}}{\mu_{f}}\right)S$$

where the differentiation with respect to η is denoted by prime, S is the constant mass flux velocity parameter. Here S>0 represents suction and S<0 represents injection, respectively, and $\sigma \left(x\right)=\sqrt{\left(n+1\right)/2}\;x^{\left(n-1\right)/2}$.

Substituting the similarity variables (7) into Equations (1)–(3), the following ordinary differential equations are obtained:

(9) $$\frac{\mu_{hnf}/\mu_{f}}{\rho_{hnf}/\rho_{f}}\;f^{\prime \prime \prime }+f\;f^{\prime }-\frac{2\;n}{n+1}{f^{\prime }}^{2}=0$$

(10) $$\frac{\mu_{hnf}/\mu_{f}}{\rho_{hnf}/\rho_{f}}\;g^{\prime \prime \prime }+f\;g”-\frac{2\;n}{n+\;1}f^{\prime }g^{\prime }=0$$

(11) $$\frac{1}{Pr}\frac{k_{hnf}/k_{f}}{{\left(\rho C_{p}\right)}_{hnf}/{\left(\rho C_{p}\right)}_{f}}\theta ”+\frac{2\;n}{n+1}\;f\theta^{\prime }=0$$

with the corresponding boundary conditions:

(12) $$\begin{array}{c} f\left(0\right)=S,\;\;\;f^{\prime }\left(0\right)=\lambda ,\;\;\;\;g\left(0\right)=0,\;\;g^{\prime }\left(0\right)=1,\;\;\theta \left(0\right)=1\;\\ f^{\prime }\left(\eta \right)\to 0,\;\;\;\;g^{\prime }\left(\eta \right)\to 0,\;\;\;\;\theta \left(\eta \right)\to 0\;\;\;\;\;\mathrm{as}\;\;\;\;\eta \to \infty \;\;\;\;\; \end{array}\}$$

where Pr is the Prandtl number, which are defined as $Pr={\left(\rho \;C_{p}\right)}_{f}/k_{f}$.

From a physical point of view we are interested in the skin friction coefficients $C_{fx}$ and $C_{fy,}$ and the Nusselt local number Nu, presented as follows:

(13) $$C_{fx}=\frac{\mu_{hnf}}{\rho_{f}}\;{\left(\frac{\partial \;u}{\partial \;z}\right)}_{z=0},\;\;\;\;C_{fy}=\frac{\mu_{hnf}}{\rho_{f}}\;{\left(\frac{\partial \;v}{\partial \;z}\right)}_{z=0},\;\;\;\;\;Nu_{x}=\frac{k_{hnf}}{k_{f}}\;{\left(-\frac{\partial \;T}{\partial \;z}\right)}_{z=0}$$

Using (7) and (13), we get

(14) $$\begin{array}{c} Re_{x}^{1/2}C_{fx}=\frac{\mu_{hnf}}{\mu_{f}}\;f^{\prime \prime }\left(0\right),\;\;\;\;Re_{x}^{1/2}C_{fy}=\frac{\mu_{hnf}}{\mu_{f}}\;g^{\prime \prime }\left(0\right),\\ \;Re_{x}^{-1/2}\;Nu_{x}=-\;\frac{k_{hnf}}{k_{f}}\;\theta^{\prime }\left(0\right) \end{array}\}$$

where $Re_{x}=u_{w}\left(x\right)\;x/\nu_{f}$ and $Re_{y}=u_{w}\left(y\right)\;y/\nu_{f}$ are the local Reynolds numbers.

We can observe that for a normal fluid (ϕ=0), Equation (9) is similar with Equation (6) from Mahapatra et al. [26], namely:

(15) $$f^{\prime \prime \prime }+f\;f^{\prime }-\frac{2\;n}{n+1}f^{\prime 2}=0$$

subject to:

(16) $$f\left(0\right)=0,\;\;\;f^{\prime }\left(0\right)=1,\;\;\;\;f^{\prime }\left(\eta \right)\to 0\;\;\;\;\mathrm{as}\;\;\;\eta \to \infty$$

However, for a regular fluid (ϕ=0) and n=1, Equation (9) reduces to:

(17) $$f^{\prime \prime \prime }+f\;f^{\prime }-f^{\prime 2}=0$$

subject to:

(18) $$f\left(0\right)=S,\;\;\;\;f^{\prime }\left(0\right)=\lambda ,\;\;\;\;f^{\prime }\left(\eta \right)\to 0\;\;\;\;\;as\;\;\;\;\eta \to \infty$$

The exact solution of the boundary value problem (17) and (18) is given by (Vajravelu and Cannon [27] or Cortell [28]), as:

(19) f(η)=S+α [1−exp(−β η)],       (β=S+α>0)

where:

(20) $$\beta =\frac{1}{2}\;(S\;\pm \;\sqrt{S^{2}+4\;\lambda })$$

Thus, we have:

(21) $$f^{\prime \prime }\left(0\right)=-\frac{\lambda }{2}\;(S\;\pm \;\sqrt{s^{2}+4\;\lambda })$$

so that it gives, as expected, $\lambda_{c}=-\frac{s^{2}}{4}$.

## 3. Discussion of the Results

Using the bvp4c solver from MATLAB programming language, Equations (9)–(11) and the conditions (12) at the boundaries are numerically solved for the value of the Prandtl number fixed to Pr=6.2 corresponding to pure water at 25 °C. In particular, bvp4c is a finite-difference code that implements the three-stage Lobatto IIIa formula (see Shampine et al. [29]). This is a collocation formula that provides a continuous solution with fourth-order accuracy. Mesh selection and error control are based on the residual of the continuous solution. The effectiveness of this solver ultimately counts on our ability to provide the algorithm with an initial prediction for the numerical result. Due to the fact that our problem may have multiple (dual) solutions, the bvp4c solver has to be provided with an initial estimate of the solution for the problem (9)–(12). The size of our mesh and the tolerance of the error are 0.001 and $10^{-9}$ respectively, ensuring accurate enough numerical results. Based on this guess value, the velocity and temperature contours are determined satisfying the boundary conditions (12) at infinity. Because the bvp4c method will converge to the first solution even for poor guesses we can use a simple initial guess for the first solution. However, for the second solution of Equations (9)–(12), it is more complicated to determine a good guess which will be adequate. Also, this convergence issue is influenced by the value of the selected parameters. In this paper we studied both stretching case sheet (λ>0) and shrinking (λ<0) case sheet. The obtained numerical results for the reduced skin friction $-f^{\prime \prime }\left(0\right)$ can be compared with those reported by Vajravelu and Cannon [27] or Cortell [28], and they are shown in Table 2. Since our results match very well we are confident that our solutions are correct and precise.

**Figure 2:**
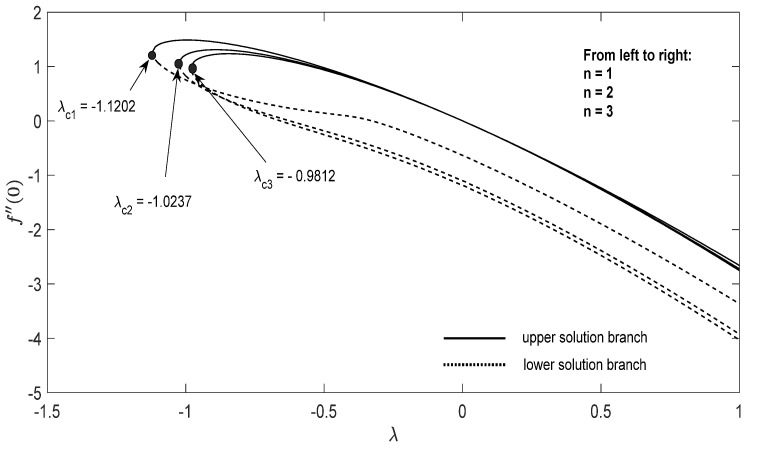
Change in $f^{\prime \prime }\left(0\right)$ with *λ* for some values of n for the hybrid nanoparticles fluid having *ϕ_1_* (Al_2_O_3_) = 0.025 and *ϕ_2_* (Cu) = 0.025 for S=2.

**Figure 3:**
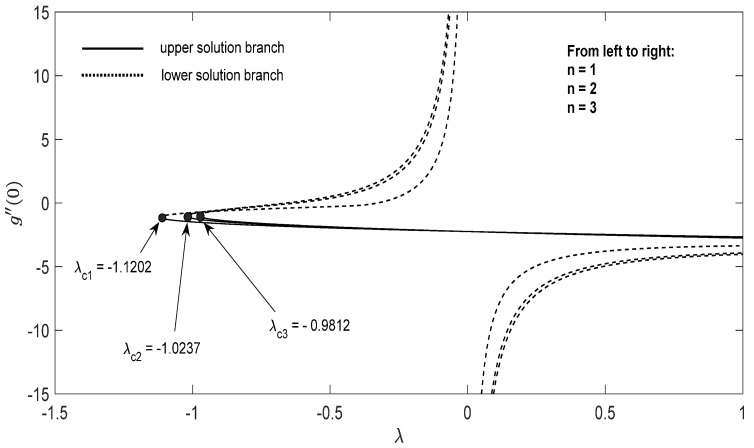
Change in $g^{\prime \prime }\left(0\right)$ with *λ* for some values of n for the hybrid nanoparticles fluid having *ϕ_1_* (Al_2_O_3_) = 0.025 and *ϕ_2_* (Cu) = 0.025 for S=2.

**Figure 4:**
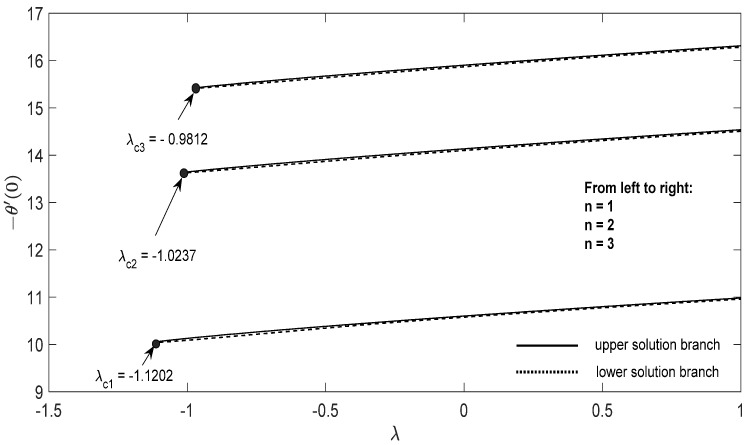
Change in $-\theta^{\prime }\left(0\right)$ with *λ* for some values of n for the hybrid nanoparticles fluid having *ϕ_1_* (Al_2_O_3_) = 0.025 and *ϕ_2_* (Cu) = 0.025 for S=2.

**Figure 5:**
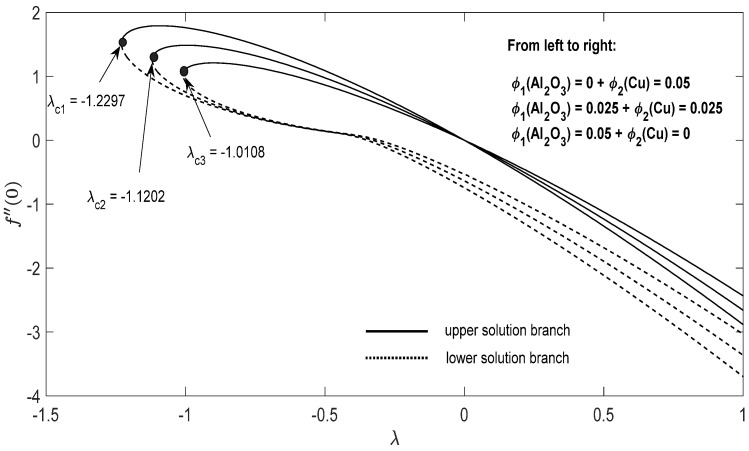
Change in $f^{\prime \prime }\left(0\right)$ with parameter *λ* for the case of hybrid nanoparticles fluid having distinct concentrations of Al_2_O_3_ and Cu nanoparticles when n=1, S=2.

**Figure 6:**
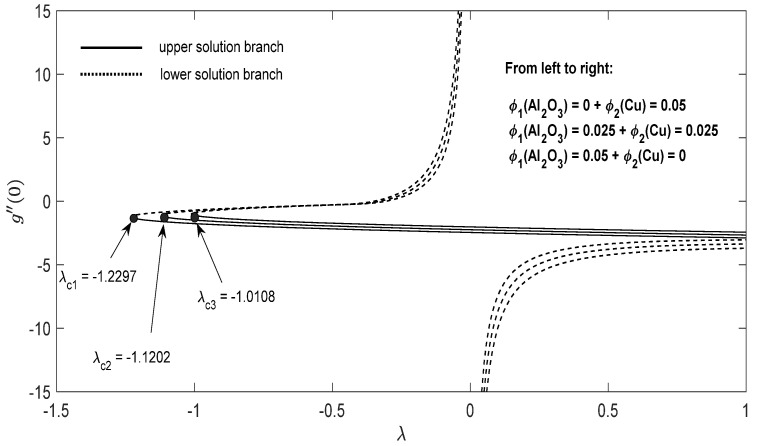
Change in $g^{\prime \prime }\left(0\right)$ with parameter *λ* for the case of hybrid nanoparticles fluid having distinct concentrations of Al_2_O_3_ and Cu nanoparticles when n=1, S=2.

**Figure 7:**
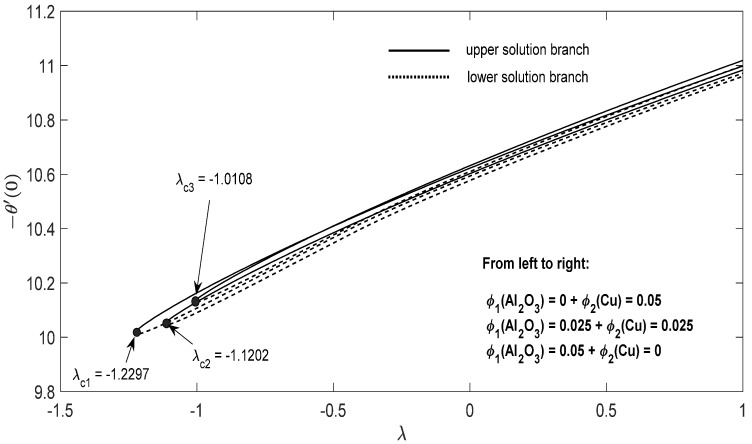
Change in $-\theta^{\prime }\left(0\right)$ with parameter *λ* for the case of hybrid nanoparticles fluid having distinct concentrations of Al_2_O_3_ and Cu nanoparticles when n=1, S=2.

**Figure 8:**
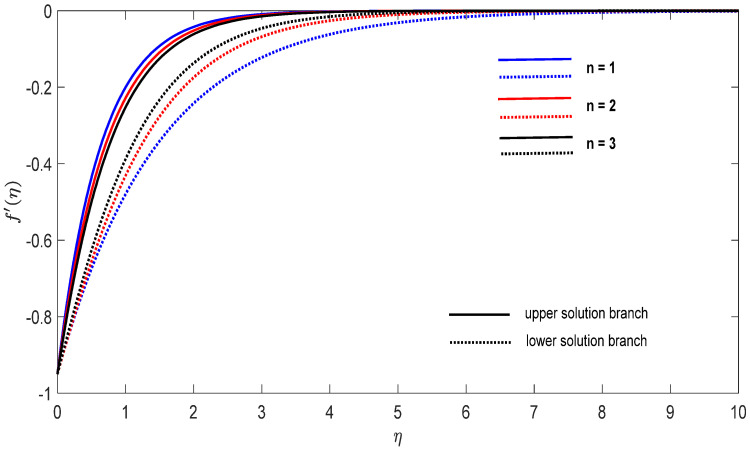
Change on the dimensionless velocity $f^{\prime }\left(\eta \right)$ for some n when λ=−0.95, S=2 for concentrations ϕ_1_ (Al_2_O_3_) = 0.025 and ϕ_2_ (Cu) = 0.025.

**Figure 9:**
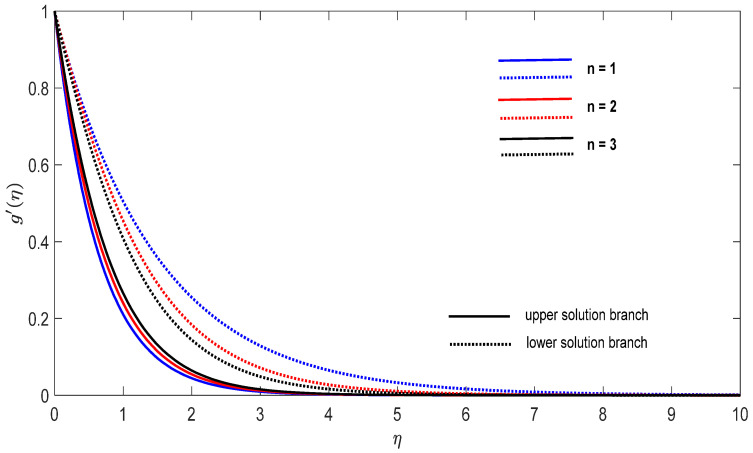
Change on the dimensionless velocity $g^{\prime }\left(\eta \right)$ for some n when λ=−0.95, S=2 for concentrations ϕ_1_ (Al_2_O_3_) = 0.025 and ϕ_2_ (Cu) = 0.025.

**Figure 10:**
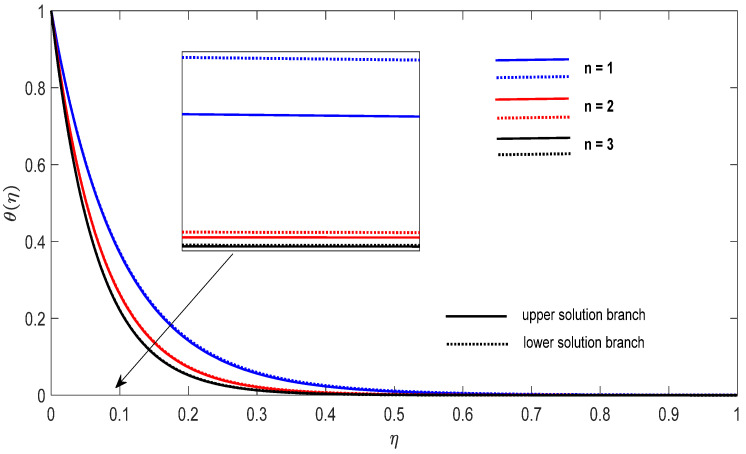
Change on the dimensionless temperature θ(η) for some n when λ=−0.95, S=2 for concentrations ϕ_1_ (Al_2_O_3_) = 0.025 and ϕ_2_ (Cu) = 0.025.

**Figure 11:**
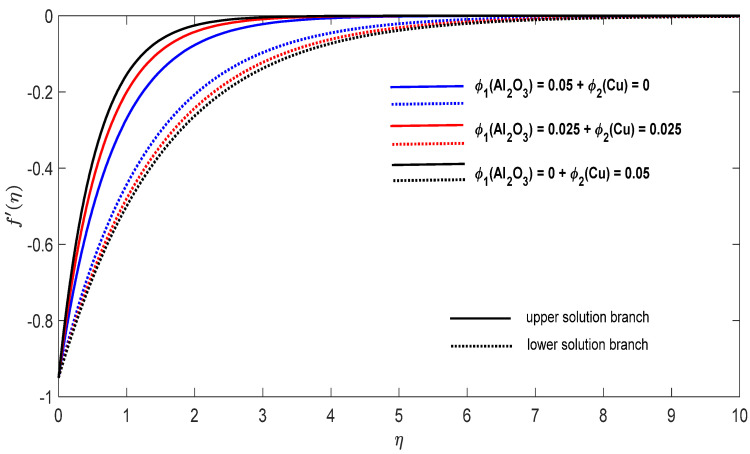
Change in the dimensionless velocity $f^{\prime }\left(\eta \right)$ for λ=−0.95, S=2 and n=1 in the case of distinct concentrations of nanoparticles.

**Figure 12:**
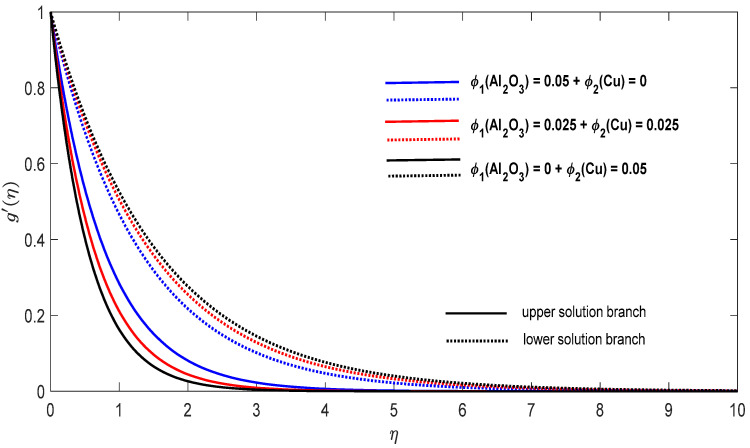
Change in the dimensionless velocity $g^{\prime }\left(\eta \right)$ for λ=−0.95, S=2 and n=1 in the case of distinct concentrations of nanoparticles.

**Figure 13:**
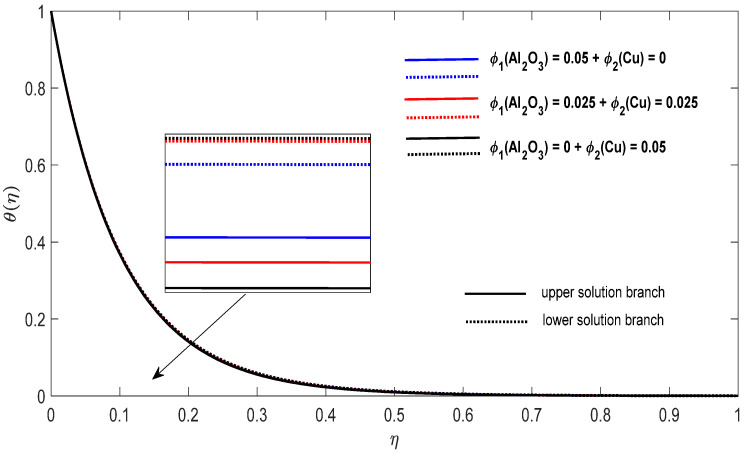
Change in the dimensionless temperature θ(η) for λ=−0.95, S=2 and n=1 in the case of distinct concentrations of nanoparticles.

Graphs that display the influence of the governing parameters on the reduced skin friction coefficients *f*′(0) and *g*′(0), the reduced local Nusselt $-\theta^{\prime }\left(0\right)$, velocity $f^{\prime }\left(\eta \right)$, $g^{\prime }\left(\eta \right)$ as well as the temperature θ(η) contours were obtained and displayed in Figure 2, Figure 3, Figure 4, Figure 5, Figure 6, Figure 7, Figure 8, Figure 9, Figure 10, Figure 11, Figure 12 and Figure 13. For numerical results we considered the non-dimensional parameter values as: Prandtl number Pr, the power-law parameter n=1–3, the stretching/shrinking parameter $\lambda_{c}\;\leq \lambda <1$ in Figure 2, Figure 3, Figure 4, Figure 5, Figure 6 and Figure 7 and λ=−0.95 in Figure 8, Figure 9, Figure 10, Figure 11, Figure 12 and Figure 13, and the constant mass flux velocity parameter S=2. Also, different nanofluid particles concentrations are considered in this paper. Figure 2, Figure 3, Figure 4, Figure 5, Figure 6 and Figure 7 show that for the studied problem (9)–(12) dual solutions (upper and lower branch solutions) exist for $\lambda_{c}\leq \lambda$ (shrinking and stretching sheet) and there is no solution in the case that $\lambda <\lambda_{c}<0$. Here $\lambda_{c}<0$ represents the critical value of λ<0 starting at the ODE’s system (9) to (12) has no solutions. We should assert that in the situation that $\lambda <\lambda_{c}<0$ the full equations (Navier–Stokes and energy) must be resolved.

Finally, Figure 8, Figure 9, Figure 10, Figure 11, Figure 12 and Figure 13 present the velocities $f^{\prime }\left(\eta \right)$ and $g^{\prime }\left(\eta \right)$ contours and the temperature θ(η) contours for λ=−0.95 (shrinking sheet situation). In these figures the upper branch solution is illustrated by solid lines, while the lower branch solution is depicted by the dotted lines, respectively. It is obvious looking at these figures that the conditions at infinity (12) are verified. This shows the correctness of our numerical results for the boundary-value problem (9)–(12). We deduce from Figure 8, Figure 9, Figure 10, Figure 11, Figure 12 and Figure 13 that the dimensionless contours for velocity and temperature of the upper branch solution show a thinner boundary layer thickness in comparison to the lower branch one.

## 4. Conclusions

Our main conclusions are the following:

- For the potential stretching and shrinking flows there exist two solutions (first/upper branch and second/lower branch), when some of the governing parameters lie in specific intervals.
- We can control the separation of the boundary layer by using lower magnitude of λ<0.
- The increment of the copper or alumina nano-sized particles solid volume concentration has a large influence on the critical value $\lambda_{c}<0$ of λ<0.
- The increase of concentration $\phi_{2}\left(Cu\right)$ raises the velocity of the fluid along the x direction for the shrinking case, while the velocity of the fluid along the y direction shows an opponent result. The increment of concentration $\phi_{1}\left(Al_{2}O_{3}\right)$ amplify the velocity of the fluid in the y direction for the shrinking case, whereas the velocity of the fluid in the x direction shows a contrary result. The above conclusions are true for the upper branch solution. For the lower branch solution the results show contrary behaviors.
- The increase in n comes along with a contraction of the temperature contours for the shrinking sheet situation for both upper and lower branch solutions.
- The increase in concentration $\phi_{1}\left(Al_{2}O_{3}\right)$ comes along with a small dilatation of the temperature contours for the shrinking case for the first solution. A contrary behavior is seen for the second solution.

## Figures and Tables

**Table 1:** Thermo-physical characteristics (Oztop and Abu-Nada [25]).

Physical Characteristics	Water	$Al_{2}O_{3}$	Cu
$\varrho \;\left(\mathrm{kg}/m^{3}\right)$	997.0	3970	8933
$C_{p}\left(J/\mathrm{kgK}\right)$	4180	765	385
k (W/mK)	0.6071	40	400

**Table 2:** Comparisons of $-f^{\prime \prime }\left(0\right)$ for some *n*, in the case $\phi_{1}=\phi_{2}=0$, S=0 and λ=1.

n	$-f^{\prime \prime }\left(0\right)$		
	Vajravelu and Cannon [27]	Cortell [28]	Present Study
0	-	0.627547	0.627554
0.2	-	0.766758	0.766837
0.5	-	0.889477	0.889543
0.75	-	0.953786	0.953956
1	1	1.0	1.0
1.5	-	1.061587	1.061600
3	-	1.148588	1.148593
5	1.1945	-	1.194487
7	-	1.216847	1.216850
10	1.2348	1.234875	1.234874
20	-	1.257418	1.257423
100	-	1.276768	1.276773

## Nomenclature

Roman letters	
a	positive constant
b	share strength constant
$C_{fx},C_{fy}$	skin friction coefficient along the *x*- and *y*- directions, respectively
$C_{p}$	specific heat at constant pressure (${\mathrm{Jkg}}^{-1}K^{-1}$)
f(η),g(η)	dimensionless stream function in the *x-* and *y*- directions, respectively (${\mathrm{ms}}^{-1}$)
k	thermal conductivity of the fluid (${\mathrm{Wm}}^{-1}K^{-1}$)
n	is the power-law stretching/shrinking parameter
$Nu_{x}$	local Nusselt number
Pr	Prandtl number
Rex,Rey	local Reynolds number in the *x-* and *y*- directions, respectively
S	suction parameter
t	time (s)
T	fluid temperature (K)
$T_{w}$	wall temperature (K)
$T_{\infty }$	ambient temperature (K)
u,v,w	velocities in the *x-*, *y*- and *z-* directions (${\mathrm{ms}}^{-1}$)
$u_{w},v_{w}$	velocities of the stretching/shrinking sheet in the x- and y- directions, respectively $({\mathrm{ms}}^{-1}$)
x,y,z	Cartesian coordinates (m)
Greek symbols	
θ	dimensionless temperature
λ	stretching/shrinking parameter
η	similarity variable
μ	dynamic viscosity of the fluid (${\mathrm{kgm}}^{-1}s^{-1}$)
ν	kinematic viscosity of the fluid ($m^{2}s^{-1}$)
ρ	fluid density (${\mathrm{kgm}}^{-3}$)
$\rho C_{p}$	heat capacity +(${\mathrm{JK}}^{-1}m^{-3}$)
$\phi_{1}$	alumina volume fractions
$\phi_{2}$	copper volume fractions
Subscripts	
f	base fluid
nf	nanofluid
hnf	hybrid nanofluid
s1	solid component for alumina (Al_2_O_3_)
s2	solid component for copper (Cu)
Superscript	
′	differentiation with respect to η
